# Supplementary material for: Triglyceride-glucose index and the risk of heart failure: Evidence from two large cohorts and a mendelian randomization analysis
Source: Cardiovasc Diabetol. 2022 Nov 3;21:229. doi: 10.1186/s12933-022-01658-7 (PMC9635212; doi:10.1186/s12933-022-01658-7)
Supplement: Supplementary file 1 — Supplementary Material 1 [file 12933_2022_1658_MOESM1_ESM.docx]

**Supplementary Material**

| **List of Supplementary Materials** | **Page** |
| --- | --- |
| Supplementary Methods | 2-3 |
| Figure S1. The flow chart of participants in the Kailuan cohort. | 4 |
| Figure S2. The flow chart of participants in the Hong Kong cohort. | 5 |
| Figure S3. Fractional polynomial curves showing the risk of heart failure across the spectrum of observed triglyceride-glucose (TyG) index in the (A) Kailuan cohort and (B) Hong Kong cohort with multivariable adjustments. | 6 |
| Figure S4. The main assumption of Mendelian randomization analysis | 7 |
| Figure S5**.** Leave-one-out sensitivity analysis of MR. | 8 |
| Table S1. International Classification of Diseases, Ninth revision (ICD-9) codes used to identify outcomes and co-morbidities. | 9-11 |
| Table S2. Heterogeneity tests and MR-Egger intercept of TyG index linked to HF. | 12 |
| Table S4. The data sources of TyG associated SNPs with confounders adjusted in MVMR analysis. | 13 |
| Table S5. Multivariable Mendelian randomization of TyG index with HF adjusting for confounders. | 14 |
| References for Supplementary Material | 15 |

**Supplementary Methods:**

**Data collection and definitions**

In the Kailuan cohort, demographic data, occupation and income information, smoking status, alcohol intake, education level, physical activity, history of cardiovascular diseases, and medication use were collected from baseline questionnaires. During biennial physical examinations, anthropometric parameters and blood pressure were measured. Detailed procedures of measurements have been described elsewhere. Blood samples were drawn from participants after an overnight fast. Serum triglyceride was measured by the enzymatic colorimetric method. Fasting blood glucose was measured by hexokinase/glucose-6-phosphate dehydrogenase method. Serum levels of high-sensitivity C-reactive protein (hs-CRP) were measured by high-sensitivity nephelometry assay. The diagnosis of diabetic mellitus was defined as FBG ≥ 7.0 mmol/L, ongoing anti-antidiabetic treatment, or any self-reported history. Hypertension was defined as systolic blood pressure (SBP) ≥ 140 mmHg or diastolic blood pressure (DBP) ≥ 90 mmHg, with physician diagnosis of hypertension, or ongoing antihypertensive treatment. Dyslipidemia was determined as total cholesterol (TC) ≥ 6.22 mmol/L, or low-density lipoprotein cholesterol (LDL-c) ≥ 4.14 mmol/L, or high-density lipoprotein cholesterol (HDL-c) ≤1.04 mmol/L, or triglycerides (TG) ≥ 2.26 mmol/L, or using lipid lowering drugs.

For the Hong Kong cohort, diagnoses of all comorbid conditions (dyslipidaemia, diabetes mellitus, hypertension, and chronic kidney disease) were first identified by ICD-9 codes as summarized in **Supplementary Table 1**. The diagnoses were further supplemented by use of medications and/or laboratory measurements: dyslipidemia was supplemented by the use of lipid-lowering medications; diabetes mellitus was supplemented by the use of any anti-diabetic medications, fasting blood glucose ≥ 7.0 mmol/L, or HbA1c >6.5%; hypertension was supplemented by the use of anti-hypertensive medications, SBP ≥ 140 mmHg, or DBP ≥ 90 mmHg; and chronic kidney disease was supplemented by baseline estimated glomerular filtration rate of <60 mL/min/1.73m2 as calculated from the 2021 Chronic Kidney Disease Epidemiology Collaboration (CKD-EPI) formula^1, 2^, wherever baseline creatinine level was available. Baseline use of selected medications was recorded, including anti-hypertensive medications, lipid-lowering medications, anti-diabetic medications, and antiplatelets. Selected laboratory measurements at baseline were recorded, including fasting blood glucose, fasting triglycerides, fasting LDL-C, fasting HDL-C, fasting total cholesterol. SBP and DBP at baseline were also recorded.

**Staged Cox regression.**

First, an unadjusted model was fitted. Second, an age and sex-adjusted model was fitted. Third, further adjusted models were fitted according to the variables included in individual cohort. For Kailuan cohort, the third model was further adjusted for education, income, physical activity, smoking status, alcohol use, diabetes mellitus, LDL-c, HDL-c, SBP, DBP, body mass index (BMI), eGFR, hs-CRP, antihypertensive drugs, antidiabetic drugs, and lipid-lowering drugs. For the cohort from Hong Kong, the third model was further adjusted for plus hypertension, diabetes mellitus, chronic kidney disease, dyslipidemia, antihypertensives, anti-diabetic drugs, and antiplatelets.

**Figure S1.** The flow chart of participants in the Kailuan Cohort.


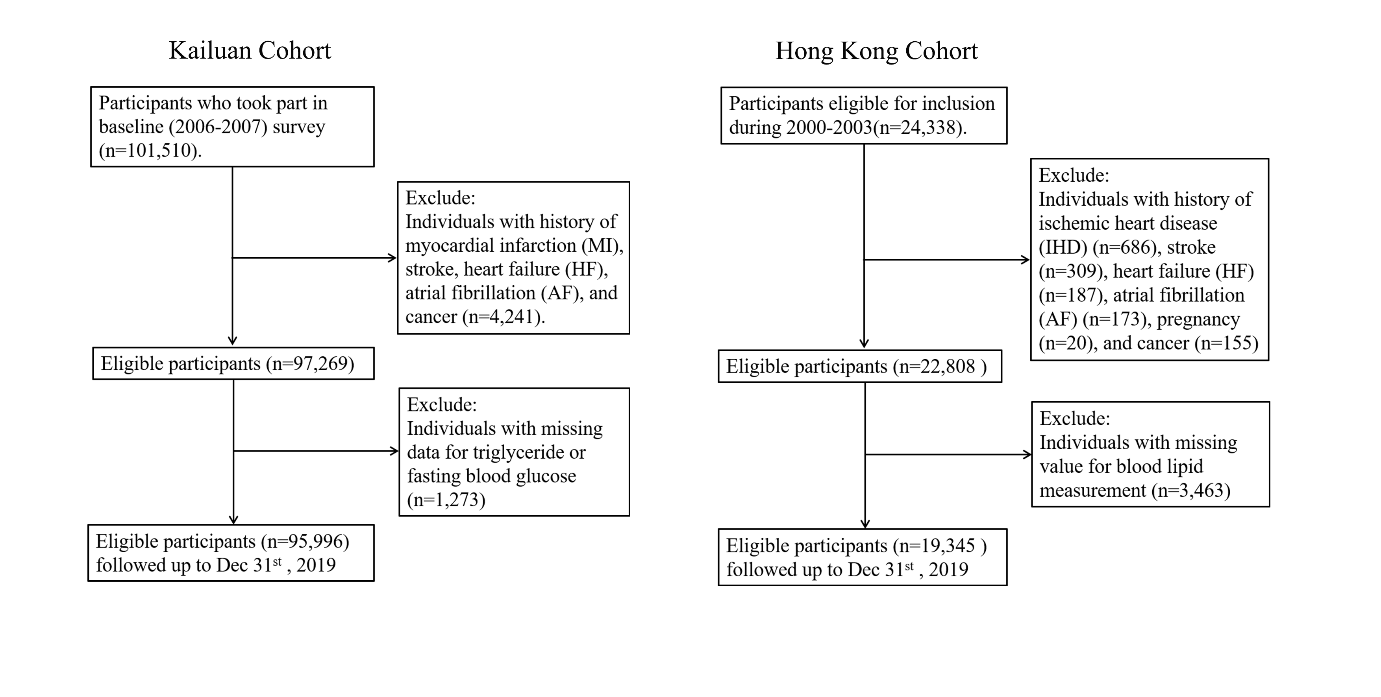


**Figure S2.** The flow chart of participants in the Hong Kong Cohort.


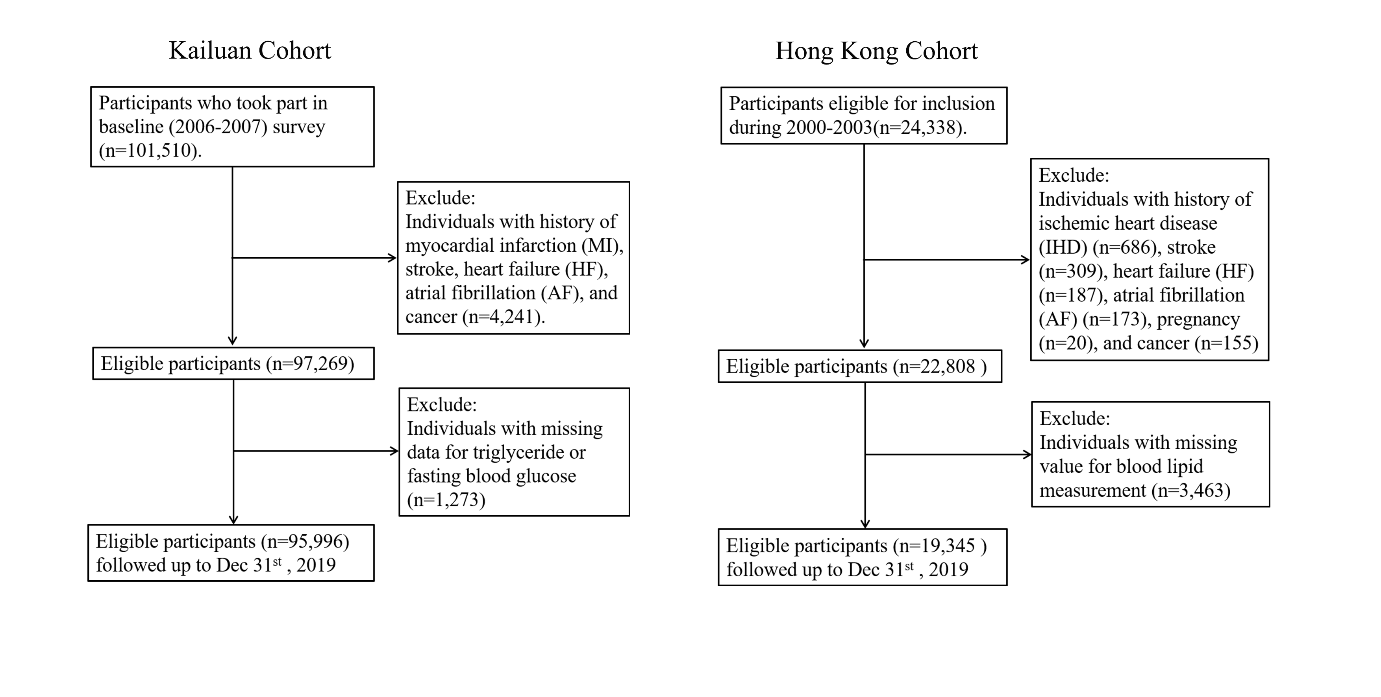


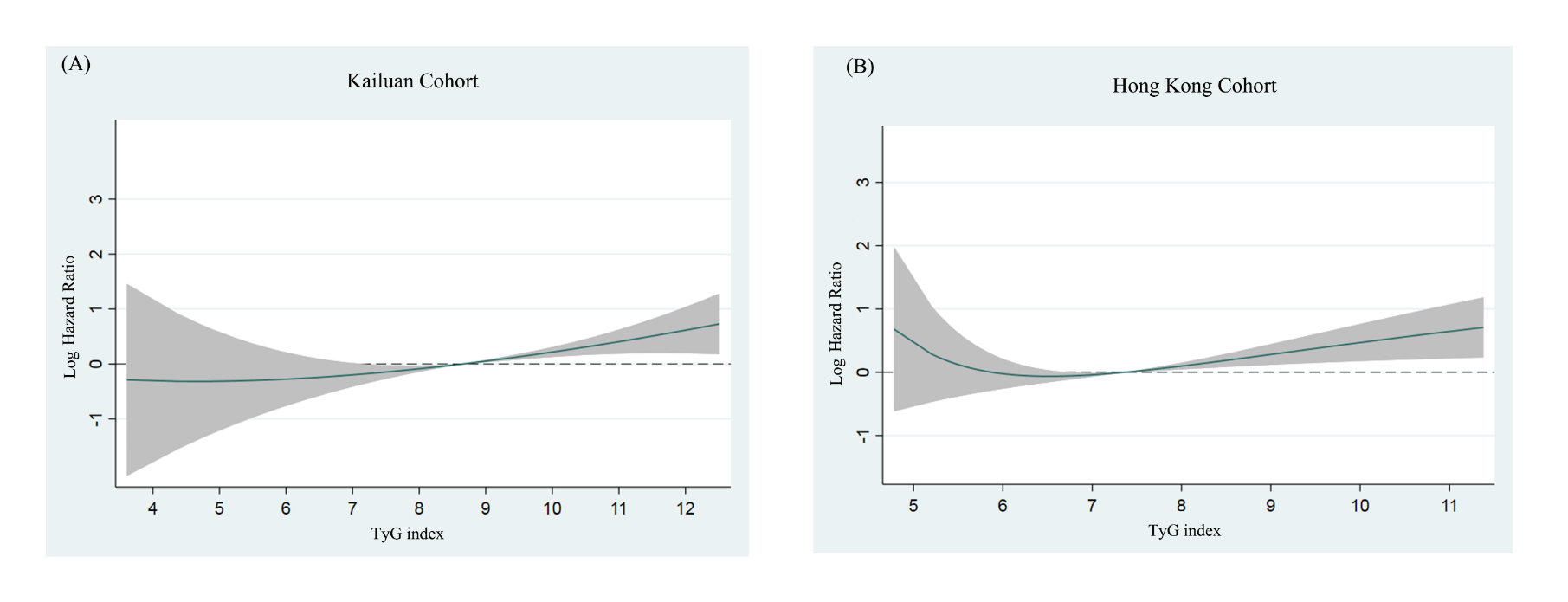
**Figure S3.** Fractional polynomial curves showing the risk of heart failure across the spectrum of observed triglyceride-glucose (TyG) index in the (A) Kailuan cohort and (B) Hong Kong cohort with multivariable adjustments.

**Figure S4**. Three main assumptions underlying Mendelian randomization study design.


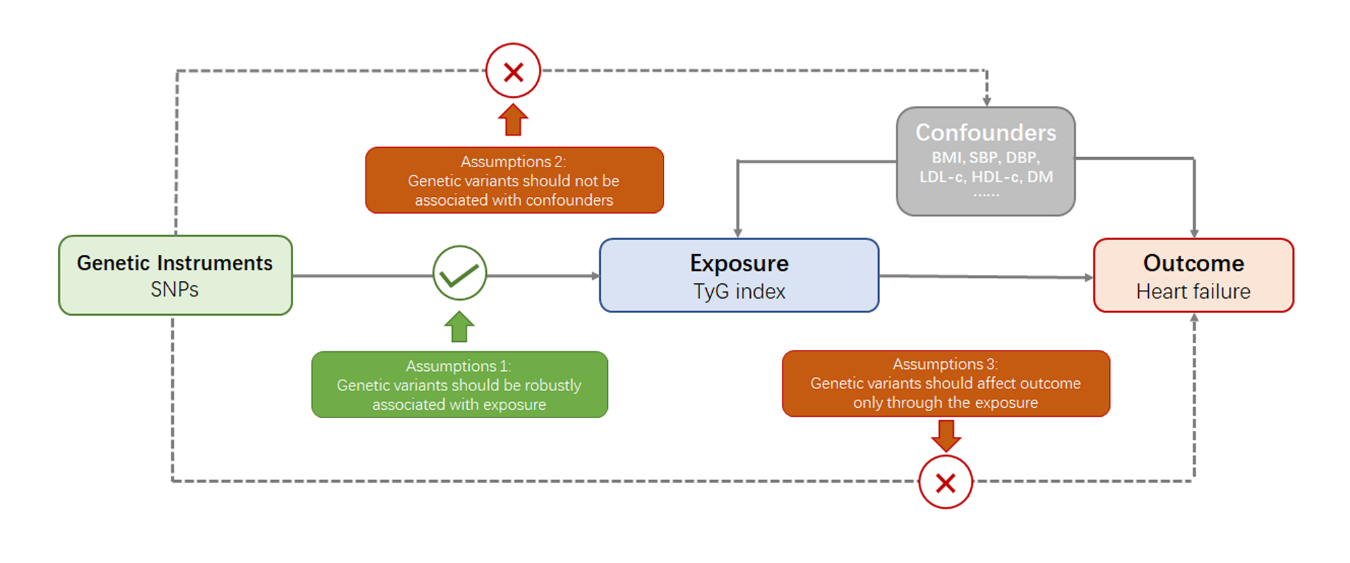


SNP: single nucleotide polymorphisms; TyG: Triglyceride-glucose; BMI: body mass index; SBP: systolic blood pressure; DBP: diastolic blood pressure; LDL-c: low-density lipoprotein cholesterol; HDL-c: high-density lipoprotein cholesterol; DM: diabetes.

**Figure S5.** Leave-one-out sensitivity analysis of MR analysis.


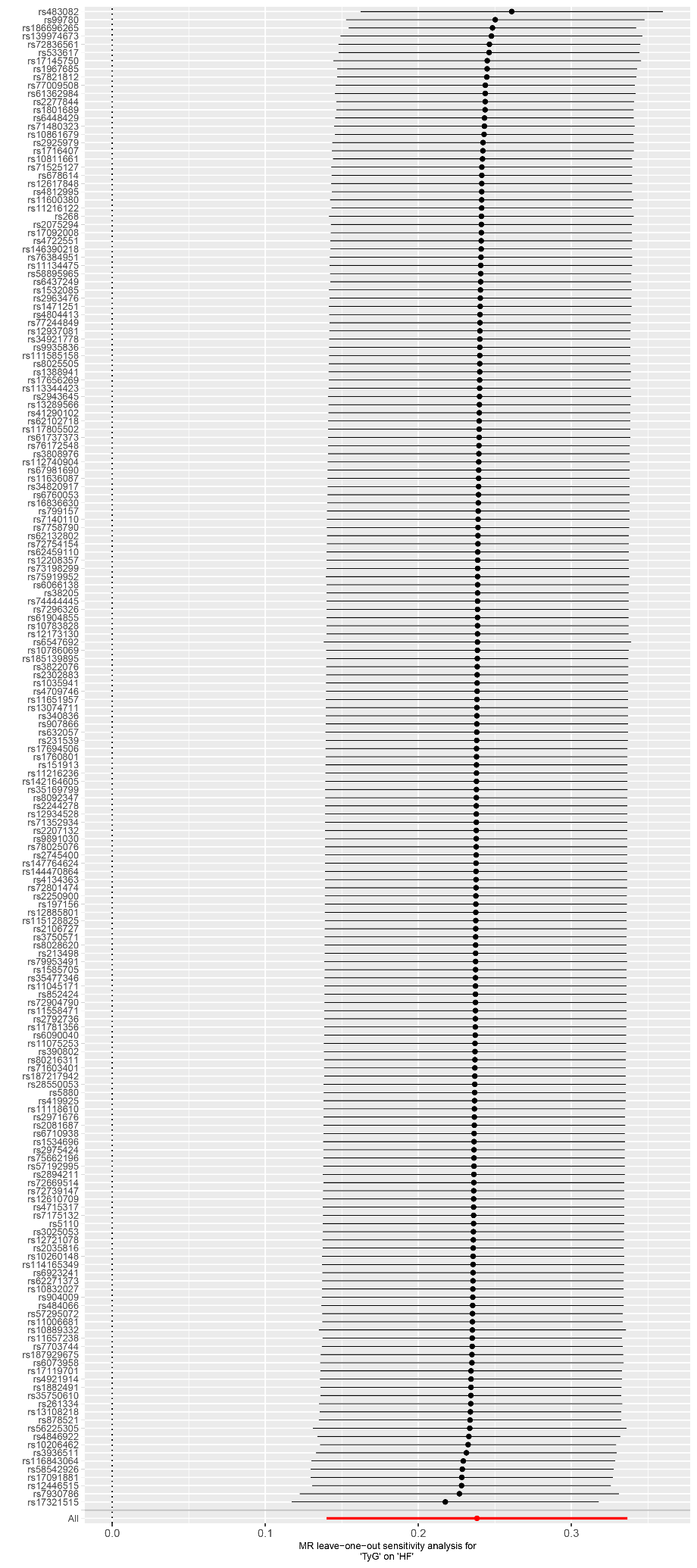


**Table S1. International Classification of Diseases, Ninth revision (ICD-9) codes used to identify outcomes and co-morbidities.**

| Heart failure | 428 428 428.1 428.2 428.2 428.21 428.22 428.23 428.3 428.31 428.32 428.33 428.4 428.4 428.41 428.42 428.43 428.9 398.91 402.01 402.11 402.91 404.01 404.03 404.11 404.13 404.91 404.93 | |  |
| --- | --- | --- | --- |
| Myocardial infarction | 410.01 410.02 410.1 410.11 410.12 410.2 410.21 410.22 410.3 410.31 410.32 410.4 410.41 410.42 410.5 410.51 410.52 410.6 410.61 410.62 410.7 410.71 410.72 410.8 410.81 410.82 410.9 410.91 410.92 | |  |
| Diabetes mellitus | 250 250.01 250.02 250.03 250.1 250.11 250.12 250.13 250.2 250.21 250.22 250.23 250.3 250.31 250.32 250.33 250.4 250.41 250.42 250.43 250.5 250.51 250.52 250.53 250.6 250.61 250.62 250.63 250.7 250.71 250.72 250.73 250.8 250.81 250.82 250.83 250.9 250.91 250.92 250.93 | |  |
| Hypertension | 401 401.1 401.9 402 402.01 402.1 402.11 402.9 402.91 403 403.01 403.1 403.11 403.9 403.91 404 404.01 404.02 404.03 404.1 404.11 404.12 404.13 404.9 404.91 404.92 404.93 405 405.01 405.09 405.1 405.11 405.19 405.9 405.91 405.99 437.2 | |  |
| Atrial fibrillation | 427.31 429.4 | |  |
| Stroke | 435 435.1 435.2 435.3 435.8 435.9 433.81 433.91 434 436 437 437.1 433.31 433.01 434.01 434.1 434.11 434.9 434.91 437.2 437.3 437.4 437.5 437.6 437.7 437.8 437.9 430 431 432 432.1 432.9 | |  |
| Ischaemic heart disease | 410.01 410.02 410.1 410.11 410.12 410.2 410.21 410.22 410.3 410.31 410.32 410.4 410.41 410.42 410.5 410.51 410.52 410.6 410.61 410.62 410.7 410.71 410.72 410.8 410.81 410.82 410.9 410.91 410.92 411 411.1 411.8 411.81 411.89 413 413.1 413.9 414 414.01 414.02 414.03 414.04 414.05 414.06 414.07 414.1 414.11 414.12 414.19 414.2 414.3 414.4 414.8 414.9 410 412 | |  |
| Dyslipidaemia | | 272.0 272.1 272.2 272.3 272.4 272.8 272.9 | |
| Chronic kidney disease | | 582 582 582.1 582.2 582.4 582.8 582.81 582.89 582.9 583 583 583.1 583.2 583.4 583.6 583.7 585 585.1 585.2 585.3 585.4 585.5 585.6 585.9 586 588 588 588.1 588.8 588.81 588.89 588.9 | |
| Cancer | | 140 140.1 140.3 140.4 140.5 140.6 140.8 140.9 141 141.1 141.2 141.3 141.4 141.5 141.6 141.8 141.9 142 142.1 142.2 142.8 142.9 143 143.1 143.8 143.9 144 144.1 144.8 144.9 145 145.1 145.2 145.3 145.4 145.5 145.6 145.8 145.9 146 146.1 146.2 146.3 146.4 146.5 146.6 146.7 146.8 146.9 147 147.1 147.2 147.3 147.8 147.9 148 148.1 148.2 148.3 148.8 148.9 149 149.1 149.8 149.9 150 150.1 150.2 150.3 150.4 150.5 150.8 150.9 151 151.1 151.2 151.3 151.4 151.5 151.6 151.8 151.9 152 152.1 152.2 152.3 152.8 152.9 153 153.1 153.2 153.3 153.4 153.5 153.6 153.7 153.8 153.9 154 154.1 154.2 154.3 154.8 155 155.1 155.2 156 156.1 156.2 156.8 156.9 157 157.1 157.2 157.3 157.4 157.8 157.9 158 158.8 158.9 159 159.1 159.8 159.9 160 160.1 160.2 160.3 160.4 160.5 160.8 160.9 161 161.1 161.2 161.3 161.8 161.9 162 162.2 162.3 162.4 162.5 162.8 162.9 163 163.1 163.8 163.9 164 164.1 164.2 164.3 164.8 164.9 165 165.8 165.9 170 170.1 170.2 170.3 170.4 170.5 170.6 170.7 170.8 170.9 171 171.2 171.3 171.4 171.5 171.6 171.7 171.8 171.9 172 172.1 172.2 172.3 172.4 172.5 172.6 172.7 172.8 172.9 173 173.01 173.02 173.09 173.1 173.11 173.12 173.19 173.2 173.21 173.22 173.29 173.3 173.31 173.32 173.39 173.4 173.41 173.42 173.49 173.5 173.51 173.52 173.59 173.6 173.61 173.62 173.69 173.7 173.71 173.72 173.79 173.8 173.81 173.82 173.89 173.9 173.91 173.92 173.99 174 174.1 174.2 174.3 174.4 174.5 174.6 174.8 174.9 175 175.9 176 176.1 176.2 176.3 176.4 176.5 176.8 176.9 179 180 180.1 180.8 180.9 181 182 182.1 182.8 183 183.2 183.3 183.4 183.5 183.8 183.9 184 184.1 184.2 184.3 184.4 184.8 184.9 185 186 186.9 187 187.1 187.2 187.3 187.4 187.5 187.6 187.7 187.8 187.9 188 188.1 188.2 188.3 188.4 188.5 188.6 188.7 188.8 188.9 189 189.1 189.2 189.3 189.4 189.8 189.9 190 190.1 190.2 190.3 190.4 190.5 190.6 190.7 190.8 190.9 191 191.1 191.2 191.3 191.4 191.5 191.6 191.7 191.8 191.9 192 192.1 192.2 192.3 192.8 192.9 193 194 194.1 194.3 194.4 194.5 194.6 194.8 194.9 195 195.1 195.2 195.3 195.4 195.5 195.8 200 200.01 200.02 200.03 200.04 200.05 200.06 200.07 200.08 200.1 200.11 200.12 200.13 200.14 200.15 200.16 200.17 200.18 200.2 200.21 200.22 200.23 200.24 200.25 200.26 200.27 200.28 200.3 200.31 200.32 200.33 200.34 200.35 200.36 200.37 200.38 200.4 200.41 200.42 200.43 200.44 200.45 200.46 200.47 200.48 200.5 200.51 200.52 200.53 200.54 200.55 200.56 200.57 200.58 200.6 200.61 200.62 200.63 200.64 200.65 200.66 200.67 200.68 200.7 200.71 200.72 200.73 200.74 200.75 200.76 200.77 200.78 200.8 200.81 200.82 200.83 200.84 200.85 200.86 200.87 200.88 201 201.01 201.02 201.03 201.04 201.05 201.06 201.07 201.08 201.1 201.11 201.12 201.13 201.14 201.15 201.16 201.17 201.18 201.2 201.21 201.22 201.23 201.24 201.25 201.26 201.27 201.28 201.4 201.41 201.42 201.43 201.44 201.45 201.46 201.47 201.48 201.5 201.51 201.52 201.53 201.54 201.55 201.56 201.57 201.58 201.6 201.61 201.62 201.63 201.64 201.65 201.66 201.67 201.68 201.7 201.71 201.72 201.73 201.74 201.75 201.76 201.77 201.78 201.9 201.91 201.92 201.93 201.94 201.95 201.96 201.97 201.98 202 202.01 202.02 202.03 202.04 202.05 202.06 202.07 202.08 202.1 202.11 202.12 202.13 202.14 202.15 202.16 202.17 202.18 202.2 202.21 202.22 202.23 202.24 202.25 202.26 202.27 202.28 202.3 202.31 202.32 202.33 202.34 202.35 202.36 202.37 202.38 202.4 202.41 202.42 202.43 202.44 202.45 202.46 202.47 202.48 202.5 202.51 202.52 202.53 202.54 202.55 202.56 202.57 202.58 202.6 202.61 202.62 202.63 202.64 202.65 202.66 202.67 202.68 202.7 202.71 202.72 202.73 202.74 202.75 202.76 202.77 202.78 202.8 202.81 202.82 202.83 202.84 202.85 202.86 202.87 202.88 202.9 202.91 202.92 202.93 202.94 202.95 202.96 202.97 202.98 203 203.01 203.02 203.1 203.11 203.12 203.8 203.81 203.82 204 204.01 204.02 204.1 204.11 204.12 204.2 204.21 204.22 204.8 204.81 204.82 204.9 204.91 204.92 205 205.01 205.02 205.1 205.11 205.12 205.2 205.21 205.22 205.3 205.31 205.32 205.8 205.81 205.82 205.9 205.91 205.92 206 206.01 206.02 206.1 206.11 206.12 206.2 206.21 206.22 206.8 206.81 206.82 206.9 206.91 206.92 207 207.01 207.02 207.1 207.11 207.12 207.2 207.21 207.22 207.8 207.81 207.82 208 208.01 208.02 208.1 208.11 208.12 208.2 208.21 208.22 208.8 208.81 208.82 208.9 208.91 208.92 196 196.1 196.2 196.3 196.5 196.6 196.8 196.9 197 197.1 197.2 197.3 197.4 197.5 197.6 197.7 197.8 198 198.1 198.2 198.3 198.4 198.5 198.6 198.7 198.8 198.81 198.82 198.89 199 199.1 | |

**Table S3. Heterogeneity tests and MR-Egger intercept of TyG index linked to HF.**

| Exposure | Outcome | Intercept | *P*^1^ | Cochran’s *Q* | *P*^2^ |
| --- | --- | --- | --- | --- | --- |
| TyG index | HF | -0.00038 | 0.81 | 290.21 | < 0.01 |

*P*^1^ Value of p for MR-Egger intercept

*P*^2^ Value of p for heterogeneity by performing inverse-variance weighted method.

TyG index, Triglyceride-glucose index; HF, Heart failure.

**Table S4. The data sources of TyG associated SNPs with confounders adjusted in MVMR analysis.**

| Phenotype | Study Type | No. of participants | PMID |
| --- | --- | --- | --- |
| BMI | Meta-analysis of GWAS | 339,224 | 25673413 |
| SBP | Meta-analysis of GWAS | 757,601 | 30224653 |
| DBP | Meta-analysis of GWAS | 757,601 | 30224653 |
| LDL-c | GWAS | 173,082 | 24097068 |
| HDL-c | GWAS | 187,167 | 24097068 |
| DM | Meta-analysis of GWAS | 110,452 | 24509480 |

BMI: body mass index; SBP: systolic blood pressure; DBP: diastolic blood pressure; LDL-c: low-density lipoprotein cholesterol; HDL-c: high-density lipoprotein cholesterol; DM: diabetes mellitus.

**Table S5. Multivariable Mendelian randomization of TyG index with HF adjusting for confounders.**

| Model | SNP number | OR (95% CI) | *P* value |
| --- | --- | --- | --- |
| Adjusted for BMI | 107 | 1.36 (1.20 – 1.53) | <0.001 |
| Adjusted for SBP | 164 | 1.20 (1.08 – 1.35) | 0.001 |
| Adjusted for DBP | 167 | 1.25 (1.12 – 1.39) | <0.001 |
| Adjusted for LDL-c | 114 | 1.22 (1.06 – 1.39) | 0.005 |
| Adjusted for HDL-c | 115 | 1.32 (1.16 – 1.49) | <0.001 |
| Adjusted for DM | 111 | 1.40 (1.25-1.57) | <0.001 |

BMI: body mass index; SBP: systolic blood pressure; DBP: diastolic blood pressure; LDL-c: low-density lipoprotein cholesterol; HDL-c: high-density lipoprotein cholesterol; DM: diabetes mellitus.

**References for Supplementary Material**

#17, #21, #22
