# Supplementary material for: Triglyceride-glucose index and the risk of heart failure: Evidence from two large cohorts and a mendelian randomization analysis
Source: Cardiovasc Diabetol. 2022 Nov 3;21:229. doi: 10.1186/s12933-022-01658-7 (PMC9635212; doi:10.1186/s12933-022-01658-7)
Supplement: Supplementary file 2 — Supplementary Material 2 [file 12933_2022_1658_MOESM2_ESM.docx]

STROBE Statement—checklist of items that should be included in reports of observational studies

|  | Item No. | Recommendation | Page  No. | Relevant text from manuscript |
| --- | --- | --- | --- | --- |
| **Title and abstract** | 1 | (*a*) Indicate the study’s design with a commonly used term in the title or the abstract | 1 | Triglyceride-glucose index and the risk of heart failure: evidence from two large cohorts and a Mendelian randomization analysis |
|  |  | (*b*) Provide in the abstract an informative and balanced summary of what was done and what was found | 3-4 | Subjects without prevalent cardiovascular diseases from the prospective Kailuan cohort (recruited during 2006-2007) and a retrospective cohort of family medicine patients from Hong Kong (recruited during 2000-2003) were followed up until December 31st, 2019 for the outcome of incident HF. Separate adjusted hazard ratios (aHRs) summarizing the relationship between TyG index and HF risk in the two cohorts were combined using a random-effect meta-analysis.  In total, 95,996 and 19,345 subjects from the Kailuan and Hong Kong cohorts were analyzed, respectively, with 2,726 cases of incident HF in the former and 1,709 in the latter. Subjects in the highest quartile of TyG index had the highest risk of incident HF in both cohorts (Kailuan: aHR 1.23 (95% confidence interval: 1.09-1.39), PTrend <0.001; Hong Kong: aHR 1.21 (1.04-1.40), PTrend =0.007; both compared with the lowest quartile). Meta-analysis showed similar results (highest versus lowest quartile: HR 1.22 (1.11-1.34), P<0.001). |
| Introduction | | | |  |
| Background/rationale | 2 | Explain the scientific background and rationale for the investigation being reported | 5-6 | Heart failure (HF) is associated with significant morbidity and mortality, with contemporary five-year survival rates of less than 50%. ……it is critically important to identify individuals at high risk of HF and to implement preventive interventions as early as possible.  The triglyceride-glucose (TyG) index, a simple, dimensionless marker derived from fasting blood triglyceride and glucose levels as measured in routine biochemical tests, has been proposed and validated as a surrogate marker of insulin resistance[10]. Previous studies have found a positive association between TyG index and the risk of various metabolic and atherosclerotic cardiovascular diseases[11, 12]. However, few studies have been conducted to investigate the association between TyG index and the risk of incident HF, and whether the association is causal remains undetermined. |
| Objectives | 3 | State specific objectives, including any prespecified hypotheses | 6 | As such, the present study aimed to assess the association between the TyG index and the risk of incident HF. |
| Methods | | | |  |
| Study design | 4 | Present key elements of study design early in the paper | 6-7 |  |
| Setting | 5 | Describe the setting, locations, and relevant dates, including periods of recruitment, exposure, follow-up, and data collection | 6-8 | Study subjects were identified from two Chinese studies, the Kailuan cohort in northern China and a territory-wide cohort in Hong Kong.  The Kailuan Study is a prospective cohort that based on a community in the Tangshan City. Details of the study has been published elsewhere[17]. In brief, a total of 101,510 subjects (aged 18 – 98 years; 81,110 males) were enrolled in the Kailuan Study at baseline (2006-2007), and received an interview of standardized questionnaires and clinical examinations at 11 hospitals responsible for health care of the community. The subjects were then followed up with repeated questionnaires, clinical and laboratory examinations every two years.  Data for the Hong Kong cohort were extracted retrospectively from the Clinical Data Analysis and Reporting System (CDARS), an administrative electronic medical records database that records the basic demographics, diagnoses, selected procedures, medication prescriptions, and selected laboratory measurements of all patients that attended public healthcare institutions in Hong Kong which serve an estimated 90% of the population[18]. Diagnoses in CDARS were recorded using International Classification of Diseases, Ninth revision (ICD-9) codes regardless of the time of data entry, as ICD-10 has not been implemented in CDARS to date. The ICD-9 codes used for identifying comorbid conditions and the outcome (HF) were summarized in Table S1. CDARS has been extensively used in prior studies and has been shown to have good diagnostic coding accuracy[19, 20]. As only retrospective, deidentified data were used, the requirement for individual patient consent has been waived. For this study, adult patients (18 years old or above) attending a family medicine clinic in Hong Kong during the years 2000-2003 with at least one set of paired FBG and fasting TG levels at baseline were included. Patients with a history of ischemic heart disease, stroke, HF, AF, or cancer were excluded, as well as those who were pregnant at the time of inclusion, and those with missing baseline low-density lipoprotein cholesterol (LDL-C), high-density lipoprotein cholesterol (HDL-C), and total cholesterol levels.  Data collection and definitions  The data collected and definitions used in this study are detailed in Supplementary Methods[17, 21, 22]. The TyG index was calculated using the following formula, ln [fasting TG (mg/dl)×FBG (mg/dl) / 2][23]. |
| Participants | 6 | (*a*) *Cohort study*—Give the eligibility criteria, and the sources and methods of selection of participants. Describe methods of follow-up  *Case-control study*—Give the eligibility criteria, and the sources and methods of case ascertainment and control selection. Give the rationale for the choice of cases and controls  *Cross-sectional study*—Give the eligibility criteria, and the sources and methods of selection of participants | 6-8 | Study subjects were identified from two Chinese studies, the Kailuan cohort in northern China and a territory-wide cohort in Hong Kong.  The Kailuan Study is a prospective cohort that based on a community in the Tangshan City. Details of the study has been published elsewhere[17]. In brief, a total of 101,510 subjects (aged 18 – 98 years; 81,110 males) were enrolled in the Kailuan Study at baseline (2006-2007), and received an interview of standardized questionnaires and clinical examinations at 11 hospitals responsible for health care of the community. The subjects were then followed up with repeated questionnaires, clinical and laboratory examinations every two years.  Data for the Hong Kong cohort were extracted retrospectively from the Clinical Data Analysis and Reporting System (CDARS), an administrative electronic medical records database that records the basic demographics, diagnoses, selected procedures, medication prescriptions, and selected laboratory measurements of all patients that attended public healthcare institutions in Hong Kong which serve an estimated 90% of the population  Figure S1 and Figure S2 |
|  |  | (*b*) *Cohort study*—For matched studies, give matching criteria and number of exposed and unexposed  *Case-control study*—For matched studies, give matching criteria and the number of controls per case |  |  |
| Variables | 7 | Clearly define all outcomes, exposures, predictors, potential confounders, and effect modifiers. Give diagnostic criteria, if applicable | 8-9 | The data collected and definitions used in this study are detailed in Supplementary Methods[17, 21, 22]. The TyG index was calculated using the following formula, ln [fasting TG (mg/dl)×FBG (mg/dl) / 2][23].  In the Kailuan cohort, all subjects were followed from the baseline examination until the date of onset of HF, date of death, or end of follow-up (December 31st, 2019), whichever came first. HF was diagnosed by experienced cardiologists in accordance with the guidelines of the European Society of Cardiology[24]. Incident HF cases were derived from the Municipal Social Insurance Institutions, hospital discharge register, and death certificates.  In the Hong Kong cohort, all patients were followed up from inclusion until the first recorded diagnosis of HF, death, or the end of follow-up (December 31st, 2019), whichever came first. HF events of both hospitalized and outpatient episodes were identified using ICD-9 codes as summarized in Table S1. |
| Data sources/ measurement | 8* | For each variable of interest, give sources of data and details of methods of assessment (measurement). Describe comparability of assessment methods if there is more than one group | 8 | Supplementary Method. |
| Bias | 9 | Describe any efforts to address potential sources of bias | 8 | Supplementary Method. |
| Study size | 10 | Explain how the study size was arrived at | 7-8 | In brief, a total of 101,510 subjects (aged 18 – 98 years; 81,110 males) were enrolled in the Kailuan Study at baseline (2006-2007).  For this study, adult patients (18 years old or above) attending a family medicine clinic in Hong Kong during the years 2000-2003 with at least one set of paired FBG and fasting TG levels at baseline were included (19,345). |

Continued on next page

| Quantitative variables | 11 | Explain how quantitative variables were handled in the analyses. If applicable, describe which groupings were chosen and why | 10 | Continuous variables were presented as mean ± standard deviation (SD) or median with interquartile range (IQR) depending on their distribution. Categorical variables were presented as frequencies and percentages. |
| --- | --- | --- | --- | --- |
| Statistical methods | 12 | (*a*) Describe all statistical methods, including those used to control for confounding | 10-11 | The Cox regression was performed with a staged approach, as detailed in Supplementary Methods. The association between the risks of HF and the observed spectrum of TyG index was also modelled and visualized using fractional polynomial curves with full multivariable adjustments. Furthermore, competing risk regression using the Fine and Gray sub-distribution model was performed to address the potentially confounding issue of competing risk, with death from any cause as the competing event. Sub-hazard ratios (SHR) with 95% CI were used as the summary statistics. Sensitivity analyses were conducted by excluding subjects with less than two-year follow-up time, and, separately, those with medications at baseline. |
|  |  | (*b*) Describe any methods used to examine subgroups and interactions | 11 | A priori subgroup analyses were performed for age (<65 vs ≥65), gender (male vs female), diabetes (yes vs no), hypertension (yes vs no), dyslipidemia (yes vs no) for both cohorts, and, for the Kailuan cohort, for obesity (yes vs no), and hs-CRP level (<1 mg/dl vs ≥1 mg/dl). |
|  |  | (*c*) Explain how missing data were addressed | 11 |  |
|  |  | (*d*) *Cohort study*—If applicable, explain how loss to follow-up was addressed  *Case-control study*—If applicable, explain how matching of cases and controls was addressed  *Cross-sectional study*—If applicable, describe analytical methods taking account of sampling strategy | N/A |  |
|  |  | (*e*) Describe any sensitivity analyses | 11 | Sensitivity analyses were conducted by excluding subjects with less than two-year follow-up time, and, separately, those with medications at baseline. |
| Results | | | | |
| Participants | 13* | (a) Report numbers of individuals at each stage of study—eg numbers potentially eligible, examined for eligibility, confirmed eligible, included in the study, completing follow-up, and analysed | 12 | Of the 101,510 subjects who took part in the Kailuan study, 95,996 subjects were analyzed after applying the exclusion criteria (Figure S1). For the Hong Kong cohort, 24,338 patients were identified for inclusion, and 19,345 patients were analyzed after applying the exclusion criteria (Figure S2). |
|  |  | (b) Give reasons for non-participation at each stage | 12 | Figure S1 and Figure S2 |
|  |  | (c) Consider use of a flow diagram | 12 | Figure S1 and Figure S2 |
| Descriptive data | 14* | (a) Give characteristics of study participants (eg demographic, clinical, social) and information on exposures and potential confounders | 12 | Table 1 and Table 2 shows the baseline characteristics of subjects according to the baseline TyG index quartiles of two cohorts. |
|  |  | (b) Indicate number of participants with missing data for each variable of interest | 12 | Figure S1 and Figure S2 |
|  |  | (c) *Cohort study*—Summarise follow-up time (eg, average and total amount) | 13 | In the Kailuan cohort, there were 2,726 cases (2.8%) of incident HF over a mean follow-up of 12.3±2.2 years, with an overall incidence rate of 2.3 (95% CI 2.2-2.4) cases per 1000 person years. In the Hong Kong cohort, there were 1,709 cases (7.0%) of incident HF over a mean follow-up of 16.2±4.3 years, with an overall incidence rate of 5.5 (95% CI 5.3-5.8) cases per 1000 person years. |
| Outcome data | 15* | *Cohort study*—Report numbers of outcome events or summary measures over time | 13 | In the Kailuan cohort, there were 2,726 cases (2.8%) of incident HF over a mean follow-up of 12.3±2.2 years, with an overall incidence rate of 2.3 (95% CI 2.2-2.4) cases per 1000 person years. In the Hong Kong cohort, there were 1,709 cases (7.0%) of incident HF over a mean follow-up of 16.2±4.3 years, with an overall incidence rate of 5.5 (95% CI 5.3-5.8) cases per 1000 person years. |
|  |  | *Case-control study—*Report numbers in each exposure category, or summary measures of exposure |  |  |
|  |  | *Cross-sectional study—*Report numbers of outcome events or summary measures |  |  |
| Main results | 16 | (*a*) Give unadjusted estimates and, if applicable, confounder-adjusted estimates and their precision (eg, 95% confidence interval). Make clear which confounders were adjusted for and why they were included | 13-14 | Table 3 and Table 4 show the associations between the TyG index, assessed both as a categorial and continuous variable, with the respective risks of incident HF in the Kailuan and Hong Kong cohorts. The cumulative incidence of incident HF for the Kailuan and the Hong Kong cohort is shown in Figures 1A and 1B, respectively. After fully adjusting for potential confounders, patients in the highest quartile of the TyG index had significantly higher risks of incident HF than those in the lowest quartile in both the Kailuan (HR 1.23 (95% CI 1.09-1.39), P <0.001) and Hong Kong (HR 1.21 (95% CI 1.04-1.40), P =0.007) cohorts. Similarly, every unit increment in the TyG index was associated with a 17% and a 13% increase in the risk of HF in the Kailuan (HR 1.17 (95% CI 1.10-1.24), P <0.001) and Hong Kong (HR 1.13 (95% CI 1.05-1.22), P<0.001) cohorts, respectively. |
|  |  | (*b*) Report category boundaries when continuous variables were categorized | 13 | Table 3 and Table 4 |
|  |  | (*c*) If relevant, consider translating estimates of relative risk into absolute risk for a meaningful time period | N/A |  |

Continued on next page

| Other analyses | 17 | Report other analyses done—eg analyses of subgroups and interactions, and sensitivity analyses | 13-14 | Competing risk regression using the Fine and Gray sub-distribution model with death from any cause as the competing event also showed positive associations between a higher TyG index and a high risk of incident HF (Tables 3 and 4). Sensitivity analyses produced consistent and similar results (Tables 3 and 4).  Results of subgroup analyses are shown in Figure 2A and Figure 2B for the Kailuan and Hong Kong cohorts, respectively. Generally, the TyG index, analyzed as a continuous variable, was positively associated with the risk of HF across various subgroups. There was significant interaction between gender and the TyG index in the Kailuan cohort (P for interaction = 0.02), but not in the Hong Kong cohort (P for interaction = 0.11). The association between TyG index and the risk of incident HF was more prominent in female subjects than in male subjects in both cohorts [HR 1.21 (95% CI 1.02 -1.47) for female vs. 1.15 (95% CI 1.08 - 1.23) for male in the Kailuan cohort, and 1.22 (95% CI 1.10 -1.64) vs. 1.05 (95% CI 0.94 - 1.17) in the Hong Kong cohort]. |
| --- | --- | --- | --- | --- |
| Discussion | | | | |
| Key results | 18 | Summarise key results with reference to study objectives | 15 | Utilizing observational data from two large Chinese cohorts and a two-sample MR analysis based on public GWAS datasets, this study demonstrated that a high TyG index was an independent and causal risk factor for incident HF in the general population. |
| Limitations | 19 | Discuss limitations of the study, taking into account sources of potential bias or imprecision. Discuss both direction and magnitude of any potential bias | 20-21 | Nonetheless, some limitations must be noted. First, we were unable to compare the predictive power of different methods for assessing insulin resistance in our observational study, since fasting insulin levels were unavailable for most subjects. Second, inherent to all observational studies, there may be residual or unmeasured confounders that we were not able to address. Nonetheless, we have included multiple important risk factors for incident HF in the multivariable regression models, and the numerous sensitivity analyses yielded consistent results which reinforced the validity of our findings.Fourth, no information was available about the subtype of incident HF. Given the different metabolic mechanisms contributing to the pathogenesis of different types of HF[48], further research in this regard is warranted. Fifth, diagnoses of the Hong Kong cohort were identified using ICD-9 codes and could not be individually adjudicated due to the retrospective, deidentified nature of the database, as well as the large sample size. Regardless, all diagnostic codes were entered by treating clinicians, who were completely independent of the authors. CDARS has also been shown to have good coding accuracy, specifically for cardiovascular outcomes. |
| Interpretation | 20 | Give a cautious overall interpretation of results considering objectives, limitations, multiplicity of analyses, results from similar studies, and other relevant evidence | 16-21 | Previous studies have found independent associations between TyG index and risks of atherosclerotic cardiovascular diseases, including myocardial infarction and ischemic stroke[23, 31]. In a recent analysis of data from the Atherosclerosis Risk in Communities (ARIC) study, Huang et al. also reported an association between higher TyG index and higher risk of incident HF in an American population, with every standard deviation’s increase in TyG index (corresponding to a TyG index of 0.6) associated with a 15% increase in risk[32]. Our study confirmed these findings in two larger cohorts from distinct geographical regions in China. Unlike the ARIC study which was restricted to subjects between the ages of 45-64 years old, our study included adult patients across the full age range. As such, our study more closely reflects real-life practice, and our findings are thus more directly generalizable.  …… |
| Generalisability | 21 | Discuss the generalisability (external validity) of the study results | 18-19 | Having derived consistent findings from two geographically distinct regions in China, our results suggest that the TyG index, as a surrogate marker of insulin resistance, may be widely applicable and prognostically useful regardless of geographical region. As subjects with prevalent major cardiovascular diseases were excluded from the present study, the analyzed cohorts had relatively low cardiovascular risks. Our results supported the TyG index as a potentially viable and effective tool for cardiovascular risk stratification in the general population. |
| Other information | |  | | |
| Funding | 22 | Give the source of funding and the role of the funders for the present study and, if applicable, for the original study on which the present article is based | 22 | This study was supported by the National Natural Science Foundation of China (No. 81970273), the Clinical Research Plan of Shenkang Hospital Development Center of Shanghai (No. SHDC2020CR4009, SHDC2020CR6012), the Shanghai Pujiang Program (21PJD057), and the Clinical Research Plan of Shanghai Municipal Health Commission (No. 202040461). |

*Give information separately for cases and controls in case-control studies and, if applicable, for exposed and unexposed groups in cohort and cross-sectional studies.

**Note:** An Explanation and Elaboration article discusses each checklist item and gives methodological background and published examples of transparent reporting. The STROBE checklist is best used in conjunction with this article (freely available on the Web sites of PLoS Medicine at http://www.plosmedicine.org/, Annals of Internal Medicine at http://www.annals.org/, and Epidemiology at http://www.epidem.com/). Information on the STROBE Initiative is available at www.strobe-statement.org.
