# Supplementary material for: Triglyceride-glucose index and the risk of heart failure: Evidence from two large cohorts and a mendelian randomization analysis
Source: Cardiovasc Diabetol. 2022 Nov 3;21:229. doi: 10.1186/s12933-022-01658-7 (PMC9635212; doi:10.1186/s12933-022-01658-7)
Supplement: Supplementary file 3 — Supplementary Material 3 [file 12933_2022_1658_MOESM3_ESM.docx]

**STROBE-MR checklist of recommended items to address in reports of Mendelian randomization studies**^1^ ^2^

| **Item No.** | **Section** | **Checklist item** | **Page No.** | **Relevant text from manuscript** |
| --- | --- | --- | --- | --- |
| 1 | **TITLE and ABSTRACT** | Indicate Mendelian randomization (MR) as the study’s design in the title and/or the abstract if that is a main purpose of the study | 1 | Triglyceride-glucose index and the risk of heart failure: evidence from two large cohorts and a Mendelian randomization analysis |
|  | **INTRODUCTION** |  |  |  |
| 2 | **Background** | Explain the scientific background and rationale for the reported study. What is the exposure? Is a potential causal relationship between exposure and outcome plausible? Justify why MR is a helpful method to address the study question | 5-6 | Previous studies have found a positive association between TyG index and the risk of various metabolic and atherosclerotic cardiovascular diseases.However, few studies have been conducted to investigate the association between TyG index and the risk of incident HF, and whether the association is causal remains undetermined.  Mendelian randomization (MR) makes use of genetic variants as instrumental variables (IVs) to generate causal estimates of the long-term effects of risk factors on outcomes. MR analysis can overcome the limitations of residual confounding and reverse causation in conventional observational studies. |
| 3 | **Objectives** | State specific objectives clearly, including pre-specified causal hypotheses (if any). State that MR is a method that, under specific assumptions, intends to estimate causal effects | 6 | As such, the present study aimed to assess the association between the TyG index and the risk of incident HF, as well as using a two-sample MR study to determine whether such associations were causal in nature. |
|  | **METHODS** |  |  |  |
| 4 | **Study design and data sources** | Present key elements of the study design early in the article. Consider including a table listing sources of data for all phases of the study. For each data source contributing to the analysis, describe the following: |  |  |
|  | a) | Setting: Describe the study design and the underlying population, if possible. Describe the setting, locations, and relevant dates, including periods of recruitment, exposure, follow-up, and data collection, when available. | 9 | TyG index-associated variants that reached genome-wide significance (P < 5 × 10-8) were retrieved from a previous GWAS. In brief, the identified GWAS included 273,368 subjects from the United Kingdom Biobank, who were aged 40-69 and free from diabetes mellitus and lipid metabolism disorders. |
|  | b) | Participants: Give the eligibility criteria, and the sources and methods of selection of participants. Report the sample size, and whether any power or sample size calculations were carried out prior to the main analysis | 9 | TyG index-associated variants that reached genome-wide significance (P < 5 × 10-8) were retrieved from a previous GWAS. In brief, the identified GWAS included 273,368 subjects from the United Kingdom Biobank, who were aged 40-69 and free from diabetes mellitus and lipid metabolism disorders.  Detail information can be found in the previous GWAS study [16]. |
|  | c) | Describe measurement, quality control and selection of genetic variants | 9 | TyG index-associated variants that reached genome-wide significance (*P* < 5 × 10-8) were retrieved from a previous GWAS. These SNPs were further pruned by linkage disequilibrium with R2 < 0.01 and those that were significantly associated with TG or glucose were also excluded. In total, 192 IVs were selected for TyG index initially.  Detail information can be found in the previous GWAS study [16] |
|  | d) | For each exposure, outcome, and other relevant variables, describe methods of assessment and diagnostic criteria for diseases | 8-10 | The TyG index was calculated using the following formula, ln [fasting TG (mg/dl)×FBG (mg/dl) / 2]  HF cases from 26 cohorts of the HERMES Consortium were identified based on the clinical diagnosis of HF of any etiology with no specific criteria for left ventricular ejection fraction. Details of subject selection were published elsewhere [26]. |
|  | e) | Provide details of ethics committee approval and participant informed consent, if relevant | 23 | All the genetic data used in this study were derived from GWAS for which ethical approval and patient consent were previously obtained. |
| 5 | **Assumptions** | Explicitly state the three core IV assumptions for the main analysis (relevance, independence and exclusion restriction) as well assumptions for any additional or sensitivity analysis | 9 | Mendelian randomization is built upon three main assumptions[25]. First, single-nucleotide polymorphisms (SNPs) selected as instrumental variables should be robustly associated with the exposure, here as TyG index. Second, the genetic instruments should not be confounded by factors affecting the exposure-outcome association. Third, genetic variants should affect outcome (HF) only through the exposure (TyG index). |
| 6 | **Statistical methods: main analysis** | Describe statistical methods and statistics used |  |  |
|  | a) | Describe how quantitative variables were handled in the analyses (i.e., scale, units, model) | 9 | The effects of the instrumental SNPs on TyG index, as a continuous variable, were acquired at the genome-wide level of significance (P < 5 × 10−8) by using linear regression adjusted for age, sex, and the top 5 genetic principal components to control population stratification. |
|  | b) | Describe how genetic variants were handled in the analyses and, if applicable, how their weights were selected | 9-11 | The effects of the instrumental SNPs on TyG index, as a continuous variable, were acquired at the genome-wide level of significance (P < 5 × 10^−8^) by using linear regression adjusted for age, sex, and the top 5 genetic principal components to control population stratification. These SNPs were further pruned by linkage disequilibrium with R2 < 0.01 and those that were significantly associated with TG or glucose were also excluded. In total, 192 IVs were selected for TyG index initially.  In the MR analysis, the summary exposure and outcome data were first harmonized, and SNPs significantly associated with incident HF were excluded. Causal effects of TyG index on HF were estimated by the inverse-variance weighted (IVW) method. |
|  | c) | Describe the MR estimator (e.g. two-stage least squares, Wald ratio) and related statistics. Detail the included covariates and, in case of two-sample MR, whether the same covariate set was used for adjustment in the two samples | 11 | Causal effects of TyG index on HF were estimated by the inverse-variance weighted (IVW) method |
|  | d) | Explain how missing data were addressed | / | / |
|  | e) | If applicable, indicate how multiple testing was addressed | 11 | Weighted median, MR-Egger, and pleiotropy residual sum and outlier (MR-PRESSO) methods were used for supplementary analyses. |
| 7 | **Assessment of assumptions** | Describe any methods or prior knowledge used to assess the assumptions or justify their validity | 9-12 | TyG index-associated variants that reached genome-wide significance (P < 5 × 10^-8^) were retrieved from a previous GWAS.  Directional pleiotropy was assessed by MR-Egger intercepts. An additional sensitivity analysis was performed by excluding any SNP significantly associated with these confounders (P < 5 × 10^-8^).  SNPs significantly associated with incident HF were also excluded (P < 5 × 10^-8^). |
| 8 | **Sensitivity analyses and additional analyses** | Describe any sensitivity analyses or additional analyses performed (e.g. comparison of effect estimates from different approaches, independent replication, bias analytic techniques, validation of instruments, simulations) | 11-12 | To test the validity of causal effects estimates, several sensitivity analyses were conducted. First, MR analysis were conducted in SNPs pruned by linkage disequilibrium with R2 < 0.001. Second, multivariable MR (MVMR) using the IVW method was conducted to further investigate the direct causal effect of TyG index on HF after adjusting for confounders including body mass index (BMI)[27], systolic blood pressure (SBP)[28], diastolic blood pressure (DBP)[28], LDL-c[29], HDL-c[29], and DM[30]. An additional sensitivity analysis was performed by excluding any SNP significantly associated with these confounders (P < 5 × 10^-8^). |
| 9 | **Software and pre-registration** |  |  |  |
|  | a) | Name statistical software and package(s), including version and settings used | 12 | The MR analyses were performed by the TwoSampleMR, MR-PRESSO and MVMR packages with R version 4.0.2. |
|  | b) | State whether the study protocol and details were pre-registered (as well as when and where) | N/A | N/A |
|  | **RESULTS** |  |  |  |
| 10 | **Descriptive data** |  |  |  |
|  | a) | Report the numbers of individuals at each stage of included studies and reasons for exclusion. Consider use of a flow diagram | 15 | Detail information can be found in the previous GWAS study [16] |
|  | b) | Report summary statistics for phenotypic exposure(s), outcome(s), and other relevant variables (e.g. means, SDs, proportions) | 15 | In Table S4. |
|  | c) | If the data sources include meta-analyses of previous studies, provide the assessments of heterogeneity across these studies | N/A | N/A |
|  | d) | For two-sample MR:  i.  Provide justification of the similarity of the genetic variant-exposure associations between the exposure and outcome samples  ii.  Provide information on the number of individuals who overlap between the exposure and outcome studies | 15 | In Table S2 |
| 11 | **Main results** |  |  |  |
|  | a) | Report the associations between genetic variant and exposure, and between genetic variant and outcome, preferably on an interpretable scale | 15 | In Table S2 |
|  | b) | Report MR estimates of the relationship between exposure and outcome, and the measures of uncertainty from the MR analysis, on an interpretable scale, such as odds ratio or relative risk per SD difference | 15 | Analysis using the IVW method demonstrated that genetic predisposition to increased TyG index was significantly associated with an increased risk of incident HF (OR 1.27, 95% CI 1.15 -1.40, P<0.001) |
|  | c) | If relevant, consider translating estimates of relative risk into absolute risk for a meaningful time period | N/A | N/A |
|  | d) | Consider plots to visualize results (e.g. forest plot, scatterplot of associations between genetic variants and outcome versus between genetic variants and exposure) | 15 | In Figure 4 and Figure S5 |
| 12 | **Assessment of assumptions** |  |  |  |
|  | a) | Report the assessment of the validity of the assumptions | 15 | Directional pleiotropy was found by MR-Egger intercept (Table S3). The association remained consistent when using complementary methods for analysis, including weighted median, MR-Egger and MRPRESSO (Figure 4). |
|  | b) | Report any additional statistics (e.g., assessments of heterogeneity across genetic variants, such as *I^2^*, Q statistic or E-value) | 15 | The Cochran’s Q statistic indicated significant heterogeneity across SNPs, while no indication of directional pleiotropy was found by MR-Egger intercept (Table S3) |
| 13 | **Sensitivity analyses and additional analyses** |  |  |  |
|  | a) | Report any sensitivity analyses to assess the robustness of the main results to violations of the assumptions | 15 | The association remained consistent when using complementary methods for analysis, including weighted median, MR-Egger and MRPRESSO (Figure 4). |
|  | b) | Report results from other sensitivity analyses or additional analyses | 15 | To verify the causal effect of TyG index on HF, we performed multivariable MR analysis by adjusting for HF risk factors, including BMI, blood pressure, and lipids. The association remained stable after adjusting for single risk factors (Table S4) and in a fully adjusted model (OR 1.20, 95% CI 1.02-1.41, P=0.03; Figure 4). Furthermore, results of the sensitivity analysis, in which 32 SNPs with potential pleiotropy were excluded, confirmed the positive association between genetically determined TyG index and HF risk (OR 1.19, 95% CI 1.05 - 1.35, P=0.01). |
|  | c) | Report any assessment of direction of causal relationship (e.g., bidirectional MR) | 15 | directional pleiotropy was found by MR-Egger intercept. |
|  | d) | When relevant, report and compare with estimates from non-MR analyses | N/A | N/A |
|  | e) | Consider additional plots to visualize results (e.g., leave-one-out analyses) | 15 | In Figure S5 |
|  | **DISCUSSION** |  |  |  |
| 14 | **Key results** | Summarize key results with reference to study objectives | 16 | Utilizing observational data from two large Chinese cohorts and a two-sample MR analysis based on public GWAS datasets, this study demonstrated that a high TyG index was an independent and causal risk factor for incident HF in the general population. |
| 15 | **Limitations** | Discuss limitations of the study, taking into account the validity of the IV assumptions, other sources of potential bias, and imprecision. Discuss both direction and magnitude of any potential bias and any efforts to address them | 19-20 | the MR analysis was restricted to patients of European descent to reduce bias from population stratification, which may limit extrapolation of our MR results to other populations. Nevertheless, given that associations between TyG index and the risk of incident HF observed in a recent report in an American cohort (the ARIC study) were comparable to our findings as observed in Chinese cohorts, the causality established by our MR analysis is likely true in Chinese population as well. Fourth, no information was available about the subtype of incident HF. Given the different metabolic mechanisms contributing to the pathogenesis of different types of HF, further research in this regard is warranted |
| 16 | **Interpretation** |  |  |  |
|  | a) | Meaning: Give a cautious overall interpretation of results in the context of their limitations and in comparison with other studies | 16 | Importantly, utilizing MR of GWAS data, we demonstrated that the association between TyG and HF was causal by nature |
|  | b) | Mechanism: Discuss underlying biological mechanisms that could drive a potential causal relationship between the investigated exposure and the outcome, and whether the gene-environment equivalence assumption is reasonable. Use causal language carefully, clarifying that IV estimates may provide causal effects only under certain assumptions | 16-17 | Although the exact underlying mechanism for the association between TyG index and HF remains to be confirmed by further molecular studies, the well-established relationship between TyG index and insulin resistance suggests that insulin resistance may at least be an important driver of such association[10]. This was further reinforced by the results from the Kailuan cohort showing that the association between TyG index and HF was independent of chronic inflammation, as well as previous studies observing associations between insulin resistance and higher risks of incident HF independent of myocardial ischaemia[33-35]. Insulin resistance may lead to excessive circulating free fatty acids and triglycerides, which induces cardiac lipotoxicity by generating toxic lipid intermediates, and decreases cardiac efficiency by increasing fatty acid oxidation[36, 37]. Insulin resistance is also associated with disturbances of the systemic metabolic and inflammatory milieu, including increased concentrations of proinflammatory cytokines, adipokines, and catecholamines, which may trigger low-grade inflammation and chronic hypercatecholaminemia that result in detrimental effects on cardiac function[38]. Furthermore, insulin resistance is involved in the maladaptive activation of the renin-angiotensin-aldosterone system, with chronic hyperinsulinaemia inducing increased release of angiotensinogen from adipose tissue and upregulation of angiotensin II receptor expression, eventually resulting in adverse cardiac remodeling and dysfunction[39]. Nonetheless, the mechanisms between insulin resistance and HF are incompletely understood to date, and remain an important area of further research. |
|  | c) | Clinical relevance: Discuss whether the results have clinical or public policy relevance, and to what extent they inform effect sizes of possible interventions | 19 | Our results supported the TyG index as a potentially viable and effective tool for cardiovascular risk stratification in the general population.  The MR results indicated that the TyG index may act as a contributor to HF. |
| 17 | **Generalizability** | Discuss the generalizability of the study results (a) to other populations, (b) across other exposure periods/timings, and (c) across other levels of exposure | 20 | the MR analysis was restricted to patients of European descent to reduce bias from population stratification, which may limit extrapolation of our MR results to other populations. Nevertheless, given that associations between TyG index and the risk of incident HF observed in a recent report in an American cohort (the ARIC study) were comparable to our findings as observed in Chinese cohorts, the causality established by our MR analysis is likely true in Chinese population as well. |
|  | **OTHER INFORMATION** |  |  |  |
| 18 | **Funding** | Describe sources of funding and the role of funders in the present study and, if applicable, sources of funding for the databases and original study or studies on which the present study is based | 22 | This study was supported by the National Natural Science Foundation of China (No. 81970273), the Clinical Research Plan of Shenkang Hospital Development Center of Shanghai (No. SHDC2020CR4009, SHDC2020CR6012), the Shanghai Pujiang Program (21PJD057), and the Clinical Research Plan of Shanghai Municipal Health Commission (No. 202040461). |
| 19 | **Data and data sharing** | Provide the data used to perform all analyses or report where and how the data can be accessed, and reference these sources in the article. Provide the statistical code needed to reproduce the results in the article, or report whether the code is publicly accessible and if so, where | 23 | The datasets used and/or analyzed during the current study are available from the corresponding author on reasonable request. |
| 20 | **Conflicts of Interest** | All authors should declare all potential conflicts of interest | 23 | These authors declare that they have no conflicts of interests. |

This checklist is copyrighted by the Equator Network under the Creative Commons Attribution 3.0 Unported (CC BY 3.0) license.

1. Skrivankova VW, Richmond RC, Woolf BAR, Yarmolinsky J, Davies NM, Swanson SA, et al. Strengthening the Reporting of Observational Studies in Epidemiology using Mendelian Randomization (STROBE-MR) Statement. JAMA. 2021;under review.

2. Skrivankova VW, Richmond RC, Woolf BAR, Davies NM, Swanson SA, VanderWeele TJ, et al. Strengthening the Reporting of Observational Studies in Epidemiology using Mendelian Randomisation (STROBE-MR): Explanation and Elaboration. BMJ. 2021;375:n2233.
